# DEA Cross-Efficiency Ranking Method Based on Grey Correlation Degree and Relative Entropy

**DOI:** 10.3390/e21100966

**Published:** 2019-10-02

**Authors:** Qin Si, Zhanxin Ma

**Affiliations:** 1 School of Mathematical Sciences, Inner Mongolia University, Hohhot 010021, China; siqin12138@163.com; 2 School of Economics and Management, Inner Mongolia University, Hohhot 010021, China

**Keywords:** data envelopment analysis, cross-efficiency, grey correlation degree, relative entropy

## Abstract

The ranking of decision-making units (DMUs) is one of the most significant issues in efficiency evaluation. However, the calculation results from the traditional Data envelopment analysis(DEA), method sometimes include multiple efficient DMUs or multiple DMUs with the same efficiency value, in which case the approach is weak in distinguishing among these DMUs. Therefore, this study proposes a DEA cross-efficiency ranking method based on the relative entropy evaluation method and the grey relational analysis method. First, the approach uses the cross-efficiency matrix as the decision matrix of multiple criteria decision-making (MCDM), and the relationship between DMU and the ideal solution is analyzed by the grey relational analysis method and the relative entropy evaluation method. Then, the degree of the criteria is determined by Shannon entropy, and the weighted grey correlation degree and the weighted relative entropy are obtained. Finally, with the comprehensive relative closeness degree between the DMU and the ideal solution, we can sort all the DMUs accordingly. In a comparative analysis, it shows that this method analyzes the similarity between DMUs and the ideal solution from the information distance and the similarity of the data sequence curve, and has certain advantages for analyzing the ranking of DMUs.

## 1. Introduction

In practical applications, the reasonable efficiency evaluation and ranking of decision-making units (DMUs)are of great significance for the comprehensive analysis of decision-makers. Data envelopment analysis (DEA), as introduced by Charnes et al. [1], is a significant method for evaluating the relative efficiency of DMUs. It can evaluate the efficiency of the DMU and identify the DMU as efficient and inefficient. However, it lacks the power to further discriminate them. To overcome this problem, Sexton et al. [2] proposed cross-efficiency analysis method, which is an effective method to distinguish the performance among all DMUs. This method evaluates the efficiency of the DMU through self-assessment and peer assessment and ranks the DMUs by the aggregation cross efficiency scores. Therefore, the cross-evaluation method is widely utilized for ranking performances of the DMUs, but there still exist some disadvantages in many cases, such as the weights are not unique or the average cross efficiency may lose the association with weights, which cannot clearly provide the rules to help decision-makers improve their performance [3,4].

Multiple criteria decision making(MCDM) is a branch of decision-making theory which has been a significant and active research field [5,6,7]. In recent years, many multiple criteria decision-making methods have been proposed, such as AHP, TOPSIS, and GRA. Although DEA and MCDM are developed independently, many studies have shown that these methods can be combined with practical applications and leverage each other’s interests [8], such as in ranking performance of DMUs [5,8,9,10].

The relative entropy evaluation method is a popular method in decision-making, which introduces relative entropy into the TOPSIS method to measure the closeness to the ideal solution [11,12]. Since the relative entropy is not the geometric distance between the two systems which improve the distinguishing of the points located on the vertical line, the relative entropy can accurately measure the degree of difference between the two systems [11]. Grey relational analysis is a very important system analysis method in grey system theory [13]. It can apply to some uncertain problems and imperfect systems [14,15]. This method reflects the close relationship between systemic factors according to the degree of similarity or closeness of the sequence curve geometry, which can reflect the internal variation of each scheme well [16,17,18].

Therefore, this study proposes a new DEA cross-efficiency ranking method based on relative entropy evaluation method and grey relational analysis method instead of the average cross efficiency. This method can analyze the cross-efficiency matrix through the perspective of multiple criteria decision-making(MCDM). First, the cross-efficiency matrix is analyzed by the two methods, the grey correlation method and the relative entropy TOPSIS method. Then the study also applies the Shannon entropy, which determines the weight of the criteria to get the weighted grey correlation degree and the weighted relative entropy between DMU and the ideal solution. Finally, we can get the results of ranking DMUs based on the comprehensive relative closeness values between DMUs and the ideal solution. This method analyzes the similarity between DMUs and the ideal solution from the information distance and the similarity of the data sequence curve, and makes full use of the evaluation information of DMU in cross-efficiency matrix, which has certain analytical advantages for the ranking of DMUs.

In the sections that follow, the related literature is reviewed in Section 2. Section 3 briefly reviews the DEA cross-efficiency evaluation. Section 4 proposes the weighted grey correlation analysis and the weighted relative entropy evaluation method, and constructs a cross-efficiency ranking method based on these methods. Section 5 analyzes two examples to illustrate the approach proposed in this study. Some conclusions of this paper are presented in Section 6.

## 2. Literature Review

Data envelopment analysis(DEA) is a non-parametric method for evaluating the efficiency of a group of homogeneous DMUs with multiple inputs and outputs [4]. Since Charnes et al. [1] developed the CCR model in 1978, DEA methods have been widely used in various fields [19,20,21]. However, there may be multiple effective units or multiple DMUs with the same efficiency value in many DEA applications, in which case the approach cannot further distinguish among these DMUs, so the overall ranking for the DMUs cannot be achieved intuitively. To solve this problem, Sexton et al. [2] proposed to introduce peer evaluation into efficiency evaluation, this method can better overcome the shortcomings of self-evaluation in the CCR model. And with the average cross-efficiency value, it provided a ranking for all DMUs. Since these advantages, the cross-efficiency evaluation has been widely implemented in various fields, such as environmental performance assessment [22], the optimal selection of suppliers [23].

However, this method still has some areas to be further improved and there are also many theoretical studies about the cross-efficiency evaluation [24]. These approaches for cross-efficiency evaluation mainly focus on the instability of cross-efficiency evaluation values. For instance, Doyle et al. [25] developed two improvement models based on benevolent and aggressive strategies. Wang et al. [26] proposed a neutral DEA model that overcomes the difficulty of the choice between the aggressive and benevolent model of Doyle et al. [25], and also achieved a full ranking for all the DMUs. Liang et al. [27] presented a game cross-efficiency approach to get a reasonable cross-efficiency value. Wu et al. [28] developed a DEA cross-efficiency model based on Pareto improvement, which makes the cross-efficiency values for the DMUs closer to Pareto optimality. Wang et al. [29] proposed a secondary goal for DEA cross-efficiency method, in which the weights are determined by the distance to the virtual ideal DMU and non-ideal DMU.

Besides the above research approaches, another research direction is an integrated method for cross-efficiency values. Wu et al. [30] considered the link between self-evaluation and peer evaluation and introduced entropy weight into cross-efficiency evaluation to eliminate the average hypothesis. Song et al. [31] proposed a variance coefficient method based on Shannon entropy to determine the weight, which improved the idea of Wu et al. [30]. Song et al. [32] proposed a cross efficiency matrix that integrates the efficiency of MAX and MIN models, and Shannon entropy is applied to efficiency aggregation. Lee et al. [33] considered that different cross-efficiency assessment models may provide different information and integrate the efficiencies for different DEA models by the entropy weight.

These studies are based on the weighted average integration research for the traditional average cross-efficiency value. In addition, there is a ranking method combined with the MCDM method instead of the average cross-efficiency scores. Many studies have shown that these methods can be combined with practical applications and leverage each other’s interests [8]. For example, Wu et al. [34] presented an improved TOPSIS method and used it to rank the cross-efficiency of DMUs. Jahanshahloo et al. [35] proposed a new super-efficient method based on the TOPSIS method to rank the cross-efficiency of DMUs and extend it to the case where data are interval. Lotfi et al. [36] proposed a new ranking method based on DEA and TOPSIS efficient decision units that only rank efficient DMUs. Rakhshan et al. [8] proposed a new TOPSIS-DEA method to rank efficient DMUs, and discussed the properties and advantages of the proposed method. An et al. [37] proposes a comprehensive ranking of DMU in combination with DEA and AHP. 

Therefore, this study proposes a new DEA cross-efficiency ranking method based on relative entropy evaluation method and grey relational analysis method. The approach combines the characteristics of the two methods to determine the relative closeness. It shows that the similarity between DMUs and the ideal solution from the information distance and the similarity of the data sequence curve, and makes full use of the evaluation information of DMU in cross-efficiency matrix, so this method has certain analytical advantages for the ranking of DMUs.

## 3. DEA Cross-Efficiency Evaluation

Suppose there are *n* DMUs to be evaluated with *m* inputs and *s* outputs, let $x_{p}={\left(x_{1p}^{},x_{2p}^{},\ldots ,x_{mp}^{}\right)}^{T}$ be the inputs vector of the *p*-th DMU, $y_{p}={\left(y_{1p}^{},y_{2p}^{},\ldots ,y_{sp}^{}\right)}^{T}$ is the outputs vector of the *p*-th DMU, and they are all positive.

For each DMU*_p_* (*p* = 1, 2, …, *n*), the efficiency value can be measured by the following CCR model,

(1) $$\left\{ \begin{array}{l} \max\sum_{r=1}^{s}\mu_{rp}^{}y_{rp}^{}\\ s.t.\sum_{r=1}^{s}\mu_{rp}^{}y_{rj}^{}-\sum_{i=1}^{m}\nu_{ip}^{}x_{ij}^{}\leq 0,j=1,2,\ldots ,n,\\ \;\sum_{i=1}^{m}\nu_{ip}^{}x_{ip}^{}=1\\ \;\nu_{ip}^{},\mu_{rp}^{}\geq 0,i=1,\ldots ,m,r=1,2,\ldots ,s, \end{array}\right.$$

where $\nu_{ip}^{}(i=1,2,\ldots ,m)$ and $\mu_{rp}^{}(r=1,2,\ldots ,s)$ denote the weights of the *m* inputs and *s* outputs, respectively, for DMU*_p_*.

After calculating model (1), $\nu_{1p}^{*},\nu_{2p}^{*},\ldots ,\nu_{mp}^{*},$ $\mu_{1p}^{*},\mu_{2p}^{*},\ldots ,\mu_{sp}^{*}$ is a group of optimal weights for DMU*_p_*. And the optimal efficiency value $\theta_{p}^{*}$ of DMU*_p_* can be obtained. For DMU*_p_*, the peer evaluation is as follows.

(2) $$\theta_{pk}=\frac{\sum_{r=1}^{s}\mu_{rk}^{*}y_{rp}^{}}{\sum_{i=1}^{m}\nu_{ik}^{*}x_{ip}^{}},k=1,2,\ldots ,n$$

where $\theta_{pk}$ is the *k*-cross efficiency of DMU*_p_* indicates that the DMU is evaluated by DMU*_k_*. Therefore, the following cross-efficiency matrix can be constructed.

(3) $$E=\left[\begin{array}{cccc} \theta_{11}^{} & \theta_{12}^{} & \cdots & \theta_{1n}^{}\\ \theta_{21}^{} & \theta_{22}^{} & \cdots & \theta_{2n}^{}\\ \vdots & \vdots & \vdots & \vdots \\ \theta_{n1}^{} & \theta_{n2}^{} & \cdots & \theta_{nn}^{} \end{array}\right],$$

According to the idea of Sexton et al. [2], the average cross-efficiency of DMU*_p_* is

(4) $$E_{p}^{cross}=\frac{1}{n}\sum_{k=1}^{n}E_{pk},p=1,2,\ldots ,n.$$

## 4. Cross-Efficiency Ranking Method Based on Grey Correlation Degree and Relative Entropy

It can be observed that the sets of weights are provided by DMUs are the criteria for ranking in cross-efficiency analysis. Therefore, ranking of DMUs by these criteria, is in fact, an MCDM problem, and each MCDM refers to making preference decisions over the available alternatives that are characterized by multiple criteria [35].

Now, suppose there are *n* DMUs, *n* evaluation criteria, and the cross-efficiency matrix is regarded as the decision matrix, thus the structure of the alternative performance matrix is depicted in Table 1. In this section, we will analyze the cross-efficiency matrix by integrating the grey relational analysis method with the relative entropy evaluation method.

### 4.1. DEA Cross-Efficiency Based on Grey Correlation Degree

The grey system theory was developed by Deng [13], which is a very typical system analysis method. The idea of the grey correlation system method is to calculate the correlation coefficient between each criterion and the best performance, and then compare the degree of correlation between standard objects and all evaluation object [16,17,18]. According to the grey correlation degree, the concrete steps for analyzing the cross- efficiency matrix are the following.

Step 1. Normalize the cross-efficiency matrix as Formula (5).

(5) $$e_{ij}^{}=\frac{\theta_{ij}}{\sqrt{\sum_{i=1}^{n}{\left(\theta_{ij}\right)}^{2}}},i=1,\ldots ,n,$$

Therefore, we can obtain the normalized data matrix as

(6) $$E^{\prime }=\left[\begin{array}{cccc} e_{11}^{} & e_{12}^{} & \cdots & e_{1n}^{}\\ e_{21}^{} & e_{22}^{} & \cdots & e_{2n}^{}\\ \vdots & \vdots & \vdots & \vdots \\ e_{n1}^{} & e_{n2}^{} & \cdots & e_{nn}^{} \end{array}\right],$$

Step 2. Determine the ideal solution as follows,

(7) $$I=\{(e_{1}^{+},e_{2}^{+},\ldots ,e_{n}^{+})\}=\{(\underset{1\leq i\leq n}{\max}e_{ij},j\in T_{1}),(\underset{1\leq i\leq n}{\min}e_{ij},j\in T_{2})\},$$

where $T_{1}$ is the set of benefit attributes, $T_{2}$ is the set of cost attributes.

Step 3. Construct the grey correlation degree matrix as Formula (8).

(8) $$\varsigma_{ij}^{+}=\frac{\underset{i}{\min}\;\underset{j}{\min}\left|e_{ij}-e_{j}^{+}\right|+\rho \underset{i}{\max}\;\underset{j}{\max}\left|e_{ij}-e_{j}^{+}\right|}{\left|e_{ij}-e_{j}^{+}\right|+\underset{i}{\min}\;\underset{j}{\min}\left|e_{ij}-e_{j}^{+}\right|},$$

where ρ is the distinguishing coefficient, the usual value range is 0~1. In most situations, take ρ=0.5. The grey correlation degree matrix can be constructed as follows.

(9) $$\varsigma =\left[\begin{array}{cccc} \varsigma_{11}^{} & \varsigma_{12}^{} & \cdots & \varsigma_{1n}^{}\\ \varsigma_{21}^{} & \varsigma_{22}^{} & \cdots & \varsigma_{2n}^{}\\ \vdots & \vdots & \vdots & \vdots \\ \varsigma_{n1}^{} & \varsigma_{n2}^{} & \cdots & \varsigma_{nn}^{} \end{array}\right],$$

Step 4. For DMU*_i_*, the grey correlation degree between it and the ideal solution can be expressed as

(10) $$\varsigma_{i}^{+}=\sum_{j=1}^{n}\omega_{j}\varsigma_{ij}^{+},i=1,2,\ldots ,n,$$

where $\omega_{j},(j=1,2,\ldots ,n)$ is the weight of *j*-the criteria.

Obviously, the larger the $\varsigma_{i}^{+}$ mean DMU_i_ and the ideal solution are highly related. Hence, the bigger the $\varsigma_{i}^{+}$ value, the better the DMU*_i_* is. And the best DMU is the one with the largest grey correlation degree to the ideal solution.

### 4.2. DEA Cross-Efficiency Based on Relative Entropy

The relative entropy evaluation method was proposed by Zhao et al. [11], which is a MCDM method that combines relative entropy [38] with TOPSIS method. The method uses the relative entropy to measure the relative distance between the evaluated scheme and the ideal scheme, and the relative closeness degree is applied to identify order relations among all schemes [11,12]. Next, we propose the weighted relative entropy evaluation method to analyze the cross-efficiency matrix, the concrete steps are as follows:

Step 1. Normalize the cross-efficiency matrix as Formula (5).

Step 2. Determine the ideal solution as Formula (7).

According to the relative entropy evaluation method [32,33], the relative entropy between the ideal solution and DMU*_i_* can be expressed as follows.

(11) $$d_{i}=\sum_{j=1}^{n}\left[e_{j}^{+}\log\frac{e_{j}^{+}}{e_{ij}}+(1-e_{j}^{+})\log\frac{1-e_{j}^{+}}{1-e_{ij}}\right],i=1,2,\ldots ,n.$$

Step3. Calculate the weighted relative entropy degree between DMU*_i_* and the ideal solution by Formula (12).

(12) $$d_{i}^{*}=\sum_{j=1}^{n}\left[e_{j}^{+}\log\frac{e_{j}^{+}}{e_{ij}}+(1-e_{j}^{+})\log\frac{1-e_{j}^{+}}{1-e_{ij}}\right]\omega_{j}^{},i=1,2,\ldots ,n,$$

where $\omega_{j},(j=1,2,\ldots ,n)$ is the weight of *j*-th criteria.

It is clearly shown that the smaller the $d_{i}^{*}$ mean the smaller distance between DMU*_i_* and the ideal solution. Therefore, the smaller the $d_{i}^{*}$ value, the better the DMU*_i_* is. And the best DMU is the one with the smallest relative entropy value to the ideal solution.

### 4.3. Weights of Criteriasbased on Entropy

In fact, each criterion provides different information in a MCDM problem. Shannon entropy is an effective measurement when applying evaluation in uncertainty decision-making processes. According to the idea of Shannon entropy [39], the following applies Shannon entropy to analyze the cross-efficiency matrix. 

Therefore, the entropy of the *j*-th criteria is defined as:

(13) $$f_{j}=-\frac{1}{\ln n}\sum_{i=1}^{n}\rho_{ij}\cdot \ln\rho_{ij},k=1,2,\ldots ,n,$$

where $\rho_{ij}^{}=\frac{\theta_{ij}}{\sum_{i=1}^{n}\theta_{ij}},i=1,\ldots ,n,j=1,2,\ldots ,n.$

Thus, the weight $\omega_{j}$ of the *j*-th criteria is calculated by Formula (14).

(14) $$\omega_{j}=\left(1-f_{j}\right)/\sum_{j=1}^{n}\left(1-f_{j}\right),j=1,2,\ldots ,n.$$

And $\sum_{j=1}^{n}\omega_{j}=1$.

Therefore, the weights set of the criteria is obtained as $\omega =(\omega_{1},\omega_{2},\ldots ,\omega_{n}).$

### 4.4. DEA Cross-Efficiency Ranking Method Based on Grey Correlation Degree and Relative Entropy

In this work, we construct a cross-efficiency method based on combining the grey relational analysis method with the relative entropy evaluation method. According to the above steps of grey relational analysis method and relative entropy evaluation method, the corresponding weighted grey correlation degree and the weighted relative entropy are calculated. 

Now, normalize them as follows.

(15) $$C_{i}^{+}=\frac{\varsigma_{i}^{+}}{\underset{1\leq i\leq n}{\max}\varsigma_{i}^{+}},\;D_{i}^{*}=\frac{\underset{1\leq i\leq n}{\min}d_{i}^{*}}{d_{i}^{*}},i=1,2,\ldots ,n,$$

As can be seen from Formula (15), the larger $C_{i}$ and $D_{i}^{*}$ mean DMU*_i_* is closer to the ideal solution.

Thus, combined determined dimensionless grey correlation degree and relative entropy by the following Formula (16).

(16) $$S_{i}^{}=\alpha C_{i}^{+}+\beta D_{i}^{*},i=1,2,\ldots ,n,$$

where α+β=1, α and β reflect the preference degree of the decision-maker to grey correlation degree and relative entropy, respectively.

Finally, determine the rank of all DMUs on the basis of their comprehensive relative closeness from the ideal solution. According to the result of $S_{i}^{}$, the bigger the comprehensive relative closeness degree is, the better the DMU*_i_* is. Hence, the best DMU is the one with the biggest comprehensive relative closeness to the ideal solution.

## 5. Example

In this section, to illustrate the method proposed in this paper, we examine two examples in order to provide a ranking for DMUs.

### 5.1. Example1

Meng et al. [40] provide a small example, which presents data for six recent road construction projects with three inputs and three outputs, as Table 2 shows (the data taken from Meng et al. [40]), where inputs are investment return period, amount of investment, network adaptability, and outputs are network structure, surroundings environmental harmonizing, society needs. Next, we will analyze the efficiency of six recent road construction projects.

After calculating the CCR model, the cross-efficiency matrix is listed in Table 3.

According to the cross-efficiency matrix, the elements in the diagonal are the CCR efficiency scores of DMUs, which can be seen as a self-evaluation. Table 3 reports DMU_2_, DMU_3_, DMU_4_ are all efficient DMUs. Thus, in order to further distinguish between these DMUs, we analyze the cross-efficiency matrix by our proposed approach.

Step 1. Normalize the cross-efficiency matrix. We can obtain the normalized matrix as follows.

$$\left[\begin{array}{cccccc} 0.3768 & 0.3521 & 0.3407 & \begin{array}{c} 0.3333 \end{array} & 0.3666 & 0.3435\\ 0.4682 & \begin{array}{c} 0.4410 \end{array} & 0.4648 & 0.4405 & 0.4637 & 0.4439\\ 0.4179 & \begin{array}{c} 0.4311 \end{array} & 0.4648 & 0.4534 & 0.4107 & 0.4439\\ \begin{array}{c} 0.3933 \end{array} & \begin{array}{c} 0.4410 \end{array} & 0.4405 & 0.4534 & 0.4133 & 0.4439\\ \begin{array}{c} 0.4091 \end{array} & \begin{array}{c} 0.3738 \end{array} & 0.3132 & 0.3303 & 0.4130 & 0.3510\\ \begin{array}{c} \begin{array}{c} 0.3769 \end{array} \end{array} & 0.4018 & 0.3997 & 0.4178 & 0.3749 & 0.4095 \end{array}\right]$$

Step 2. Determine the ideal solution as Formula (7).

$$I=(e_{1}^{+},e_{2}^{+},e_{3}^{+},e_{4}^{+},e_{5}^{+},e_{6}^{+})=(0.4682,0.4410,0.4648,0.4534,0.4637,0.4439)$$

Step 3. Construct the grey correlation matrix between DMU and the ideal solution. According to the grey correlation analysis, we can obtain the grey correlation matrix by Formula (8) as follows.

$$\left[\begin{array}{cccccc} 0.4532 & 0.4603 & 0.3791 & \begin{array}{c} 0.3870 \end{array} & 0.4385 & 0.4302\\ 1 & \begin{array}{c} 1 \end{array} & 1 & 0.8543 & 1 & 1\\ 0.6010 & \begin{array}{c} 0.8845 \end{array} & 1 & 1 & 0.5885 & 1\\ \begin{array}{c} 0.5029 \end{array} & \begin{array}{c} 1 \end{array} & 0.7577 & 1 & 0.6010 & 1\\ \begin{array}{c} 0.5619 \end{array} & \begin{array}{c} 0.5229 \end{array} & 0.3333 & 0.3812 & 0.5992 & 0.4494\\ \begin{array}{c} 0.4536 \end{array} & 0.6590 & 0.5379 & 0.6807 & 0.4607 & 0.6877 \end{array}\right]$$

Step 4. Calculate the weights set of criteria. The weight $\omega_{j}$ of the *j*-th criteria is calculated by Formula (13) and (14), so we can get the weights set as follows.

$$\omega =(\omega_{1}^{},\omega_{2}^{},\omega_{3}^{},\omega_{4}^{},\omega_{5}^{},\omega_{6}^{})=(0.0825,0.1025,0.3137,0.2510,0.0847,0.1656).$$

Step 5. Calculate the grey correlation degree between the DMU and the ideal solution is as follows.

$$\varsigma_{1}^{+}=0.4090,\varsigma_{2}^{+}=0.9634,\varsigma_{3}^{+}=0.9204,\varsigma_{4}^{+}=0.8492,\varsigma_{5}^{+}=0.4261,\varsigma_{6}^{+}=0.5975,$$

From the results of the grey correlation degree, $\varsigma_{2}^{+}$ is the largest of them, which denotes DMU_2_ is highly relevant to the ideal solution, $\varsigma_{1}^{+}$ is the smallest of them, showing that the correlation between DMU_1_ and the ideal solution is small. 

Step 6. Calculate the relative entropy between DMU and the ideal solution. According to Formula (12), the weighted relative entropy between each DMU and the ideal solution is

$$d_{1}^{*}=0.01148,d_{2}^{*}=0.00004,d_{3}^{*}=0.00040,d_{4}^{*}=0.00077,d_{5}^{*}=0.01255,d_{6}^{*}=0.00301,$$

From the values of the relative entropy, $d_{2}^{*}$ is the smallest of them, which shows that DMU_2_ is closer to the ideal solution than other DMUs. $d_{5}^{*}$ is the biggest of them, showing that DMU_5_ is farther away from the ideal solution than others.

Step 7. Normalize $\varsigma_{i}^{+}$ and $d_{i}^{*}$ according to Formula (15).

$$C_{1}^{+}=0.4245,C_{2}^{+}=1,C_{3}^{+}=0.9553,C_{4}^{+}=0.8814,C_{5}^{+}=0.4422,C_{6}^{+}=0.6201,$$

$$D_{1}^{*}=0.0032,D_{2}^{*}=1,D_{3}^{*}=0.0911,,D_{4}^{*}=0.0481,D_{5}^{*}=0.0029,D_{6}^{*}=0.0123.$$

Further, by integrating grey correlation degree with relative entropy by Formula(16),we can get the results as follows.

$$S_{1}^{+}=0.2139,S_{2}^{+}=1,S_{3}^{+}=0.5232,S_{4}^{+}=0.4648,S_{5}^{+}=0.2226,S_{6}^{+}=0.3162.$$

Through the calculation of grey relative degree and relative entropy, first, it can be found that the effective unit is further distinguished, DMU_2_ is the most relevant to the ideal solution, and DMU_3_ and DMU_4_ are inferior to DMU_2_. Then, with the TOPSIS results as shown in Table 4, it indicates that the efficiency trends based on relative entropy evaluation method and TOPSIS are consistent, but the efficiency change based on relative entropy is more obvious, which is more convincing. Combined with the grey correlation degree, the data similarity analysis can fully reflect the data situation change and geometric similarity. Finally, it can be concluded that all DMUs are ranked as DMU_2_ > DMU_3_ > DMU_4_ > DMU_6_ > DMU_5_ > DMU_1_.

In order to assess the ranking merits of our proposed method, we apply different methods to rank DMUs. All the ranking results are listed in Table 4.

Table 4 reports that the CCR efficiencies, the rankings provided by the three different models and our proposed method, from which it is seen that there are three efficient DMUs in the CCR efficiency that cannot be further discriminated, where as our proposed approach provides the ranking result of DMUs. And the ranking result is identical to the three methods, the average cross-efficiency, benevolent method, and our proposed method, the ranking is DMU_2_ > DMU_3_>DMU_4_ > DMU _6_> DMU_5_ > DMU_1_.

However, the TOPSIS method by Wu et al. [26] results in a different ranking for some DMU in the six DMUs, such as the worst DMU is DMU_5_ instead of DMU_1_. By comparing the results, we find that the closeness results based on relative entropy are the same as TOPSIS, and DMU_1_ is closer to the ideal solution than DMU_5_. However, by analyzing the grey correlation degree, it is found that the DMU_5_ is more related to the ideal solution from the similarity of the sequence curve. Moreover, as shown in Table 3, the DMU_5_ is better than the DMU_1_ under various standards. Therefore, with comprehensive information, DMU_5_ is preferable to DMU_1_.

### 5.2. Example 2

Shang and Sueyoshi [41] provide an example, where they describe data for the technology of manufacturing systems with two inputs and four outputs, as Table 5 shows (the data taken from Shang and Sue Yoshi [41], Wu et al. [30]). Inputs are the annual operating and depreciation cost (in units of $100,000), the floor space requirements of each specific system (in thousands of square feet). Outputs are the improvements in qualitative benefits (%), work in the process reduced (10), the average number of tardy jobs reduced (%) and the average yield increased (100) [30]. Next, we will analyze the efficiency of these DMUs.

The cross-efficiency matrix is calculated by the CCR model, as shown in Table 6.

The cross-efficiency matrix shows that the elements in the diagonal are the CCR efficiency scores of DMUs, which can be seen as a self-evaluation. Table 6 shows DMU_1_, DMU_2_, DMU_4_, DMU_5_, DMU_6_, DMU_7_, DMU_9_ are all efficient DMUs. It is impossible to achieve a full ranking for all the DMUs. Thus, in order to get the ranking of all DMUs, we analyze the cross-efficiency matrix by our proposed approach.

Step 1. Normalize the cross-efficiency matrix. We can obtain the normalized matrix as follows.

$$\left[\begin{array}{cccccccccccc} 0.3213 & 0.3252 & 0.3191 & 0.3098 & 0.2577 & 0.2387 & 0.2491 & 0.3086 & 0.2323 & 0.2532 & 0.3185 & 0.3098\\ 0.3078 & 0.3252 & 0.3061 & 0.3049 & 0.2440 & 0.2332 & 0.2400 & 0.3014 & 0.2131 & 0.2333 & 0.3078 & 0.3049\\ 0.3155 & 0.2894 & 0.3134 & 0.2952 & 0.3625 & 0.3142 & 0.3379 & 0.2928 & 0.2876 & 0.2996 & 0.2941 & 0.2952\\ 0.2961 & 0.3252 & 0.2957 & 0.3098 & 0.3318 & 0.3126 & 0.3238 & 0.3086 & 0.2961 & 0.3168 & 0.2854 & 0.3098\\ 0.3213 & 0.3133 & 0.3191 & 0.3098 & 0.3902 & 0.3332 & 0.3608 & 0.3086 & 0.3109 & 0.3312 & 0.2952 & 0.3098\\ 0.2938 & 0.2767 & 0.2972 & 0.2997 & 0.3225 & 0.3640 & 0.3497 & 0.2970 & 0.3379 & 0.3151 & 0.3039 & 0.2997\\ 0.3172 & 0.2868 & 0.3191 & 0.3098 & 0.3482 & 0.3640 & 0.3608 & 0.3086 & 0.3478 & 0.3312 & 0.3185 & 0.3098\\ 0.2959 & 0.2964 & 0.2958 & 0.2944 & 0.2500 & 0.2584 & 0.2573 & 0.2967 & 0.2750 & 0.2847 & 0.3009 & 0.2944\\ 0.1198 & 0.2093 & 0.1347 & 0.2242 & 0.0084 & 0.2666 & 0.1588 & 0.2323 & 0.3478 & 0.2812 & 0.2140 & 0.2242\\ 0.2412 & 0.2394 & 0.2426 & 0.2464 & 0.2249 & 0.2488 & 0.2409 & 0.2572 & 0.3195 & 0.3158 & 0.2493 & 0.2464\\ 0.3136 & 0.3001 & 0.3116 & 0.2951 & 0.2438 & 0.2300 & 0.2381 & 0.2934 & 0.2220 & 0.2365 & 0.3131 & 0.2951\\ 0.2572 & 0.2510 & 0.2556 & 0.2482 & 0.2871 & 0.2521 & 0.2694 & 0.2451 & 0.2247 & 0.2385 & 0.2418 & 0.2482 \end{array}\right]$$

Step 2. Determining the ideal solution as follows,

$$\begin{array}{c} I=(e_{1}^{+},e_{2}^{+},e_{3}^{+},e_{4}^{+},e_{5}^{+},e_{6}^{+},e_{7}^{+},e_{8}^{+},e_{9}^{+},e_{10}^{+},e_{11}^{+},e_{12}^{+})\\ =(0.3213,0.3252,0.3191,0.3098,0.3902,0.3640,0.3608,0.3086,0.3478,0.3312,0.3185,0.3098). \end{array}$$

Step 3. Construct the grey correlation degree matrix between DMU and the ideal solution. 

According to the grey correlation analysis, we can calculate the correlation degree between each DMU and the ideal solution as Formula (8) and construct the grey correlation degree matrix as follows.

$$\left[\begin{array}{cccccccccccc} 1 & 1 & 1 & 1 & 0.5903 & 0.6036 & 0.6310 & 1 & 0.6231 & 0.7099 & 1 & 1\\ 0.9338 & 1 & 0.9632 & 0.9754 & 0.5662 & 0.5933 & 0.6126 & 0.9236 & 0.5863 & 0.6610 & 0.9469 & 0.9754\\ 0.9706 & 0.8422 & 0.9714 & 0.9290 & 0.8733 & 0.7931 & 0.8930 & 0.9236 & 0.7604 & 0.8579 & 0.8870 & 0.9290\\ 0.8835 & 1 & 0.8910 & 1 & 0.7658 & 0.7876 & 0.8378 & 1 & 0.7869 & 0.9297 & 0.8524 & 1\\ 1 & 0.9413 & 1 & 1 & 1 & 0.8609 & 1 & 1 & 0.8380 & 1 & 0.8915 & 1\\ 0.8739 & 0.7973 & 0.8974 & 0.9498 & 0.7381 & 1 & 0.9455 & 0.9427 & 0.9508 & 0.9221 & 0.9290 & 0.9498\\ 0.9791 & 0.8326 & 1 & 1 & 0.8197 & 1 & 1 & 1 & 1 & 1 & 1 & 1\\ 0.8827 & 0.8687 & 0.8914 & 0.9254 & 0.5766 & 0.6437 & 0.6485 & 0.9413 & 0.7239 & 0.8041 & 0.9159 & 0.9254\\ 0.4864 & 0.6222 & 0.5087 & 0.6904 & 0.3333 & 0.6622 & 0.4859 & 0.7145 & 1 & 0.7923 & 0.6464 & 0.6904\\ 0.7045 & 0.6898 & 0.7141 & 0.7508 & 0.5358 & 0.6236 & 0.6144 & 0.7878 & 0.8709 & 0.9254 & 0.7341 & 0.7508\\ 0.9614 & 0.8836 & 0.9625 & 0.9289 & 0.5659 & 0.5875 & 0.6088 & 0.9261 & 0.6027 & 06683 & 0.9726 & 0.9289\\ 0.7485 & 0.7201 & 0.7505 & 0.7561 & 0.6492 & 0.6303 & 0.6764 & 0.7504 & 0.6079 & 0.6730 & 0.7136 & 0.7561 \end{array}\right]$$

Step 4. Calculate the weights set of all criteria. The weight $\omega_{j}$ of the *j*-th criteria is calculated by Formulas (13) and (14), so we can get the weights set as follows.

$$\begin{array}{c} \omega =(\omega_{1}^{},\omega_{2}^{},\omega_{3}^{},\omega_{4}^{},\omega_{5}^{},\omega_{6}^{},\omega_{7}^{},\omega_{8}^{},\omega_{9}^{},\omega_{10}^{},\omega_{11}^{},\omega_{12}^{})\\ =(0.1014,0.0356,0.0843,0.0233,0.4111,0.0626,0.1077,0.0187,0.0666,0.0360,0.0296,0.0233) \end{array}$$

Step 5. Calculate the grey correlation degree between each DMU and the ideal solution. The weighted grey correlation degrees between each DMU and the ideal solution by Formula (10) are

$$\begin{array}{l} \varsigma_{1}^{+}=0.7315,\varsigma_{2}^{+}=0.6993,\varsigma_{3}^{+}=0.8833,\varsigma_{4}^{+}=0.8309,\varsigma_{5}^{+}=0.9752,\varsigma_{6}^{+}=0.8462,\\ \varsigma_{7}^{+}=0.9178,\varsigma_{8}^{+}=0.7076,\varsigma_{9}^{+}=0.5048,\varsigma_{10}^{+}=0.6443,\varsigma_{11}^{+}=0.6985,\varsigma_{12}^{+}=0.6790. \end{array}$$

From the results of the grey correlation degree, $\varsigma_{5}^{+}$ is the largest of them indicate that DMU_5_ is more similar to the ideal solution, $\varsigma_{9}^{+}$ is the smallest of them indicate that the similarity between DMU_9_ and the ideal solution is smaller than others.

Step 6. Calculate the relative entropy between each DMU and the ideal solution. According to the relative entropy, the weighted relative entropy between each DMU and the ideal solution is as follows.

$$\begin{array}{l} d_{1}^{*}=0.0112,d_{2}^{*}=0.0140,d_{3}^{*}=0.0009,d_{4}^{*}=0.0020,d_{5}^{*}=0.0002,d_{6}^{*}=0.0021,\\ d_{7}^{*}=0.0007,d_{8}^{*}=0.0111,d_{9}^{*}=0.2324,d_{10}^{*}=0.0169,d_{11}^{*}=0.0139,d_{12}^{*}=0.0091. \end{array}$$

From the score of the relative entropy, $d_{5}^{*}$ is the smallest of them, which shows that DMU_5_ is closer to the ideal solution than other DMUs. $d_{9}^{*}$ is the biggest of them, showing that the similarity between DMU_9_ and the ideal solution is smaller than others.

Step 7. Normalize $\varsigma_{i}^{+}$ and $d_{i}^{*}$ according to Formula (15).

$$\begin{array}{l} C_{1}^{+}=0.7501,C_{2}^{+}=0.7171,C_{3}^{+}=0.9057,C_{4}^{+}=0.8520,C_{5}^{+}=1.0000,C_{6}^{+}=0.8677,\\ C_{7}^{+}=0.9411,C_{8}^{+}=0.7256,C_{9}^{+}=0.5177,C_{10}^{+}=0.6607,C_{11}^{+}=0.7163,C_{12}^{+}=0.6962. \end{array}$$

$$\begin{array}{l} D_{1}^{*}=0.0151,D_{2}^{*}=0.0121,D_{3}^{*}=0.1944,D_{4}^{*}=0.0854,D_{5}^{*}=1.0000,D_{6}^{*}=0.0817,\\ D_{7}^{*}=0.2289,D_{8}^{*}=0.0153,D_{9}^{*}=0.0007,D_{10}^{*}=0.0100,D_{11}^{*}=0.0121,D_{12}^{*}=0.0185. \end{array}$$

By combining determined grey correlation degree and relative entropy by Formula (16), we can get the results as follows.

$$\begin{array}{l} S_{1}^{+}=0.3826,S_{2}^{+}=0.3646,S_{3}^{+}=0.5501,S_{4}^{+}=0.4687,S_{5}^{+}=1.0000,S_{6}^{+}=0.4747,\\ S_{7}^{+}=0.5850,S_{8}^{+}=0.3704,S_{9}^{+}=0.2592,S_{10}^{+}=0.3353,S_{11}^{+}=0.3642,S_{12}^{+}=0.3574. \end{array}$$

According to the calculation of grey relative degree and relative entropy, first, it can be found that the effective unit is further distinguished, DMU_5_ is the most relevant to the ideal solution, and DMU_1_, DMU_2_, DMU_4_, DMU_6_, DMU_7_, DMU_9_ are inferior to DMU_5_. Then, with the TOPSIS results, as shown in Table 4, it reports that the efficiency trends based on relative entropy and TOPSIS evaluation are consistent, but the efficiency change based on relative entropy is more obvious, which is more convincing. In addition, it can be observed that from the perspective of relative entropy, $d_{4}^{*}=0.0020$, $d_{6}^{*}=0.0021,$ the closeness of the two DMUs is similar. However, the degree of discrimination between DMU_6_ and DMU_4_ is large by grey correlation analysis, and DMU_6_ is obviously closer to the ideal solution than DMU_4_. Therefore, the evaluation result obtained from the comprehensive closeness is obviously improved, and the gap between DMU_4_ and DMU_6_ is increased. 

Finally, after comprehensive consideration, the ranking of all the DMUs is DMU_5_ > DMU_7_ >DMU_3_ > DMU_6_ > DMU_4_ > DMU_1_ > DMU_8_ > DMU_2_ > DMU_11_ > DMU_12_ > DMU_10_ > DMU_9_. 

In order to assess the ranking merits of our proposed method, we apply different methods to rank DMUs, the ranking results of DMUs are listed in Table 7.

Table 7 shows that seven DMUs were identified as efficient DMUs by the CCR efficiency scores, which cannot be further discriminated. It also appears that the ranking results of DMUs from these methods are different. Interestingly, the results show that the ranking results of several DMUs remain relatively stable, such as DMU_8_, DMU_9_, DMU_11_. Therefore, it is clear that DMU_9_ is an efficient DMU by the CCR efficiency scores, where as all the ranking results in Table 7 unanimously indicate that DMU_9_ performs worst of the 12 DMUs. 

In addition, the ranking result of our proposed method is closer to those of the TOPSIS method (Wu et al. [26]). By comparing the results of the two methods, we find that the order of some DMUs is different, such as DMU_10_ and DMU_12_.The rank of DMU_12_ is higher than DMU_10_ in the relative entropy and grey correlations. Furthermore, as shown in Table 6, DMU_12_ is better than DMU_10_ under most of the standards. Therefore, considering the comprehensive information, DMU_12_ is superior to DMU_10_.

## 6. Conclusions

This paper proposes a DEA cross-efficiency ranking method based on grey correlation degree and relative entropy evaluation method. This method considers the efficiency value ordering problem of decision-making unit as a MCDM problem. First, the relationship between DMU and the ideal solution are analyzed by the grey relational analysis method and the relative entropy evaluation method. Then, the study also applies the Shannon entropy, which determines the weights of criteria to get the weighted grey correlation and the weighted relative entropy between DMU and the ideal solution. With the comprehensive relative closeness degree of each DMU, we can sort all the DMUs accordingly. As seen from the results of two examples, the approach combines the characteristics of the two methods to determine the relative closeness, which makes the analysis problem more comprehensive. Moreover, considering the mutual evaluation information about DMU through Shannon entropy, it can accurately reflect the actual situation, so that the method can offer an effective full ranking of DMUs. 

## Figures and Tables

**Table 1:** Structure of the cross-efficiency matrix as the alternative performance.

	Criterion	The Criteria of DMU_1_	The Criteria of DMU_2_	…	The Criteria of DMU*_n_*
Alternative					
DMU_1_		$\theta_{11}^{}$	$\theta_{12}^{}$	…	$\theta_{1n}^{}$
DMU_2_		$\theta_{21}^{}$	$\theta_{22}^{}$	…	$\theta_{2n}^{}$
⋮		⋮	⋮	⋮	⋮
DMU*_n_*		$\theta_{n1}^{}$	$\theta_{n2}^{}$	…	$\theta_{nn}^{}$

**Table 2:** Input and output data of decision-making units (DMUs).

DMU	Inputs			Output		
	*x*_1_	*x*_2_	*x*_3_	*y*_1_	*y*_2_	*y*_3_
1	9.1	21	86	0.67	0.865	82
2	7.05	13.7	68	0.695	0.85	82
3	5.85	10.5	73.5	0.71	0.82	78.5
4	4.75	9.5	80	0.715	0.84	86
5	9.25	29.4	82.4	0.65	0.9	88.5
6	5.75	14.7	80.5	0.695	0.81	78.5

**Table 3:** The cross-efficiency matrix.

DMU	1	2	3	4	5	6
1	0.8047	0.7985	0.7330	0.7352	0.7907	0.7738
2	1.0000	1.0000	1.0000	0.9715	1.0000	1.0000
3	0.8925	0.9776	1.0000	1.0000	0.8857	1.0000
4	0.8400	1.0000	0.9479	1.0000	0.8915	1.0000
5	0.8738	0.8475	0.6738	0.7286	0.8907	0.7908
6	0.8050	0.9111	0.8599	0.9216	0.8087	0.9225

**Table 4:** The ranking results of DMUs.

DMU	CCR	Average	Aggressive	Benevolent	TOPSIS	$S_{i}^{*}$
1	0.804650 (6)	0.772630 (6)	0.693390 (6)	0.789990 (6)	0.003179 (5)	0.213873 (6)
2	1.000000 (1)	0.995250 (1)	0.926070 (3)	1.000000 (1)	0.000004 (1)	1.000000 (1)
3	1.000000 (1)	0.959290 (2)	0.934010 (2)	0.955550 (2)	0.000280 (2)	0.523184 (2)
4	1.000000 (1)	0.946550 (3)	0.953610 (1)	0.955240 (3)	0.000680 (3)	0.464766 (3)
5	0.890660 (5)	0.800860 (5)	0.676450 (5)	0.840180 (5)	0.003406 (6)	0.222587 (5)
6	0.922450 (4)	0.871440 (4)	0.792570 (4)	0.880120 (4)	0.001451 (4)	0.316201 (4)

**Table 5:** Input and output data of DMUs.

DMU	Inputs			Outputs		
	*x*_1_	*x*_2_	*y*_1_	*y*_2_	*y*_3_	*y*_4_
1	17.02	5.0	42	45.3	14.2	30.1
2	16.46	4.5	39	40.1	13.0	29.8
3	11.76	6.0	26	39.6	13.8	24.5
4	10.52	4.0	22	36.0	11.3	25.0
5	9.50	3.8	21	34.2	12.0	20.4
6	4.79	5.4	10	20.1	5.0	16.5
7	6.21	6.2	14	26.5	7.0	19.7
8	11.12	6.0	25	35.9	9.0	24.7
9	3.67	8.0	4	17.4	0.1	18.1
10	8.93	7.0	16	34.3	6.5	20.6
11	17.74	7.1	43	45.6	14.0	31.1
12	14.85	6.2	27	38.7	13.8	25.4

**Table 6:** The cross-efficiency matrix.

DMU	1	2	3	4	5	6	7	8	9	10	11	12
1	1.000	1.000	1.000	1.000	0.661	0.656	0.691	1.000	0.668	0.764	1.000	1.000
2	0.958	1.000	0.959	0.984	0.625	0.641	0.665	0.977	0.613	0.704	0.966	0.984
3	0.982	0.890	0.982	0.953	0.929	0.863	0.937	0.949	0.827	0.905	0.924	0.953
4	0.922	1.000	0.927	1.000	0.850	0.859	0.898	1.000	0.851	0.956	0.896	1.000
5	1.000	0.963	1.000	1.000	1.000	0.915	1.000	1.000	0.894	1.000	0.927	1.000
6	0.914	0.851	0.932	0.967	0.826	1.000	0.969	0.962	0.972	0.951	0.954	0.967
7	0.987	0.882	1.000	1.000	0.892	1.000	1.000	1.000	1.000	1.000	1.000	1.000
8	0.921	0.911	0.927	0.950	0.641	0.710	0.713	0.961	0.791	0.860	0.945	0.950
9	0.373	0.644	0.422	0.724	0.022	0.732	0.440	0.753	1.000	0.849	0.672	0.724
10	0.751	0.736	0.760	0.795	0.576	0.683	0.668	0.833	0.919	0.954	0.783	0.795
11	0.976	0.923	0.977	0.953	0.625	0.632	0.660	0.951	0.638	0.714	0.983	0.953
12	0.800	0.772	0.801	0.801	0.736	0.692	0.747	0.794	0.646	0.720	0.759	0.801

**Table 7:** The ranking results of DMUs.

DMU	CCR	Average	Aggressive	Benevolent	TOPSIS	$S_{i}^{*}$
1	1.00000 (1)	0.86992 (6)	0.85193 (3)	0.95498 (5)	0.00006 (6)	0.38258 (6)
2	1.00000 (1)	0.83976 (8)	0.83906 (4)	0.93555 (6)	0.00009 (8)	0.36456 (8)
3	0.98237 (9)	0.92441 (5)	0.77714 (5)	0.92452 (8)	0.00005 (5)	0.55009 (3)
4	1.00000 (1)	0.92991 (4)	0.85686 (2)	0.98118 (2)	0.00002 (3)	0.46870 (5)
5	1.00000 (1)	0.97497 (2)	0.87828 (1)	0.97697 (3)	0.00001 (1)	1.00000 (1)
6	1.00000 (1)	0.93890 (3)	0.72753 (8)	0.95560 (4)	0.00003 (4)	0.47474 (4)
7	1.00000 (1)	0.98014 (1)	0.75866 (6)	0.98786 (1)	0.00001 (2)	0.58503 (2)
8	0.96143 (10)	0.85669 (7)	0.72654 (9)	0.93078 (7)	0.00006 (7)	0.37042 (7)
9	1.00000 (1)	0.61283 (12)	0.57374 (12)	0.74874 (12)	0.00115 (12)	0.25920 (12)
10	0.95355 (11)	0.77118 (10)	0.63194 (11)	0.81473 (10)	0.00050 (10)	0.33534 (11)
11	0.98314 (8)	0.83198 (9)	0.73947 (7)	0.90770 (9)	0.00011 (9)	0.36420 (9)
12	0.80117 (12)	0.75585 (11)	0.66812 (10)	0.77344 (11)	0.00058 (11)	0.35738 (10)
